# The relevance of AMP-activated protein kinase in insulin-secreting β cells: a potential target for improving β cell function?

**DOI:** 10.1007/s13105-019-00706-3

**Published:** 2019-11-05

**Authors:** Tomasz Szkudelski, Katarzyna Szkudelska

**Affiliations:** Department of Animal Physiology and Biochemistry, Poznan University of Life Sciences, Wolynska 35, 60-637 Poznan, Poland

**Keywords:** AMPK, Insulin secretion, Pancreatic β cells, Glucose

## Abstract

AMP-activated protein kinase (AMPK) is present in different kinds of metabolically active cells. AMPK is an important intracellular energy sensor and plays a relevant role in whole-body energy homeostasis. AMPK is activated, among others, in response to glucose deprivation, caloric restriction and increased physical activity. Upon activation, AMPK affects metabolic pathways leading to increased formation of ATP and simultaneously reducing ATP-consuming processes. AMPK is also expressed in pancreatic β cells and is largely regulated by glucose, which is the main physiological stimulator of insulin secretion. Results of in vitro studies clearly show that glucose-induced insulin release is associated with a concomitant inhibition of AMPK in β cells. However, pharmacological activation of AMPK significantly potentiates the insulin-secretory response of β cells to glucose and to some other stimuli. This effect is primarily due to increased intracellular calcium concentrations. AMPK is also involved in the regulation of gene expression and may protect β cells against glucolipotoxic conditions. It was shown that in pancreatic islets of humans with type 2 diabetes, AMPK is downregulated. Moreover, studies with animal models demonstrated impaired link between glucose and AMPK activity in pancreatic islet cells. These data suggest that AMPK may be a target for compounds improving the functionality of β cells. However, more studies are required to better elucidate the relevance of AMPK in the (patho)physiology of the insulin-secreting cells.

## Introduction

Adenosine monophosphate-activated protein kinase (AMPK, EC 2.7.11.31) belongs to the family of serine/threonine kinases. AMPK occurs as a heterotrimer, consisting of a catalytic α-subunit and regulatory β- and γ-subunits. Each subunit possesses also isoforms (α1, α2, β1, β2, γ1, γ2, γ3), making a total of 12 possible heterotrimer combinations. The activation of AMPK is due to an increase in the intracellular AMP:ATP ratio and phosphorylation of Thr172 on the activation loop of the α-subunit [24]. The binding of both AMP and ADP to the γ-subunit is competitively inhibited by ATP, which indicates that AMPK is a sensor of AMP/ATP or ADP/ATP ratios. AMPK has also an inhibitory site at Ser485 of the α1 subunit [8, 19, 64].

The AMPK system is present in different kinds of cells and is an important intracellular energy sensor. It undergoes activation, among others, in response to hypoxia, ischemia, glucose deprivation and also under conditions of caloric restriction or increased physical activity [8]. AMPK affects metabolic pathways and phosphorylates several intracellular proteins, including other enzymes and thereby regulates processes associated with energy metabolism. Upon activation, AMPK usually shifts intracellular metabolic pathways toward increased formation of ATP and simultaneously inhibits ATP-consuming processes. However, some effects are tissue-specific [8, 19]. In liver, AMPK activation results in the inhibition of gluconeogenesis and promotion of lipolysis. In skeletal muscles, AMPK increases, among others, intracellular glucose transport and metabolism, and also influences glycogen metabolism. Moreover, induction of AMPK leads to the inhibition of fatty acid synthesis and to augmented mitochondrial β-oxidation. Major long-term effect, resulting from upregulation of AMPK in muscle tissue, involves promotion of mitochondrial biogenesis. This beneficial effect is associated with reduced intramuscular lipid accumulation and an improvement in insulin action [55, 64]. AMPK is also involved in the regulation of metabolism of white adipose tissue cells. Adipocytes not only store energy for other kinds of cells, but also secrete adipokines and thereby have numerous regulatory functions. An induction of AMPK in adipocytes usually results in the inhibition of fatty acid synthesis, increased β-oxidation and the inhibition of lipolysis [8, 70, 76].

AMPK is highly expressed in metabolically active tissues and, affecting intracellular pathways, plays a relevant role in whole-body energy homeostasis. Accumulating evidence indicates that dysregulation of the AMPK system is associated with metabolic disorders, metabolic syndrome, insulin resistance and also type 2 diabetes [10, 58, 75]. Moreover, the expression and/or action of AMPK is abnormal in obesity and insulin resistance [8, 78]. It is also known that chronic low-grade inflammation, which is strongly linked with metabolic diseases, downregulates AMPK in multiple tissues. These effects have been shown not only in studies with animal models, but also in humans [8, 10, 58]. It was also revealed that induction of AMPK limits inflammatory processes [10]. In parallel with these findings, insulin-sensitising drugs, which are commonly used in humans with type 2 diabetes, are known to upregulate AMPK in liver, adipose tissue and skeletal muscle. This effect is observed in the case of metformin, thiazolidinediones and some other compounds. The action of these agents is similar to effects resulting from exercise [8, 58]. Moreover, some naturally occurring compounds, which are capable of exerting beneficial effects in humans with metabolic disorders, act partially via AMPK [20, 23, 68]. Given the role of AMPK in energy homeostasis, its physiological and pharmacological modulation in tissues is very helpful in preventing and treating conditions associated with energy imbalance and insulin resistance [10].

Apart from influencing insulin action, in vitro studies show that AMPK plays also a regulatory role in the process of insulin release. Precise regulation of insulin secretion is a relevant question, since abnormal supply of this hormone is associated with various hormonal and metabolic disturbances. Hypersecretion of insulin may lead to hypoglycemia and to dangerous neuroglucopenia. However, impaired release and/or action of this hormone is associated with diabetes. Given that the number of humans with type 2 diabetes and with metabolic disorders worldwide is still increasing [26], regulatory function of AMPK in both insulin secretion and action should be thoroughly explained. This is a relevant question in the context of the therapeutic potential of AMPK modulators.

Under physiological conditions, insulin release from pancreatic β cells is tightly controlled. Regulation of this process is complex, and β cells are affected by plenty of factors. Glucose is the primary physiological stimulator of insulin synthesis and release. The insulinotropic action of glucose is strongly dependent on its metabolism. Glucose metabolism is associated with changes in intracellular energy status, and also ionic events, which finally leads to the exocytosis of insulin. Apart from glucose, some amino acids and free fatty acids have also a modulatory role [30, 38, 45, 54]. Moreover, the insulin-secretory capacity of β cells is largely promoted by gut-derived incretin hormones, i.e. glucagon-like peptide-1 (GLP-1) and glucose-dependent insulinotropic peptide (GIP). Occurring after a meal, increased release of these hormones augments the insulin-secretory response of pancreatic β cells to glucose and to some other nutrients. The relevance of GIP and GLP-1 in the process of insulin secretion is well established. It is assessed that incretins are responsible for more than a half of circulating insulin [11].

The strong dependency of the insulin secretion process on energy status of β cells rises a question about the potential regulatory involvement of AMPK, an intracellular energy sensor, in the release of this hormone [40, 77]. AMPK have been implicated in regulating many processes related to biology of β cell. These effects have been recently broadly characterised by Rourke et al. [57]. Our review focus on results of in vitro studies, using isolated pancreatic islets, β cells and isolated pancreas, showing the link between AMPK and insulin secretion. AMPK in β cells is influenced by various anti-diabetic agents [57], which action involve different mechanisms, not only related to AMPK. In the present review, effects resulting from more selective pharmacological activation of AMPK are shown. Moreover, some other aspects addressing AMPK and (patho)physiology of β cells related to insulin secretion are also described.

## The link between AMPK and insulin secretion

AMPK is expressed in rodent and human pancreatic islets and has been shown to play a role in the process of insulin secretion [32, 65]. The phosphorylation/activity of AMPK in islet cells is well established to be largely affected by glucose. Moreover, there is a close link between hormone secretion and the energy status of β cells. Intracellular glucose transport and metabolism with the formation of ATP is a prerequisite for the induction of insulin output. Importantly, pancreatic β cells are very well adapted to get maximum energy from glucose. The first step of this is intracellular glucose transport. At low, non-stimulatory concentrations, this transport is limited. However, elevated glucose levels are associated with its rapid transport via low affinity glucose transporter GLUT1 (in humans) or GLUT2 (in rodents). This is followed by glucose phosphorylation, which is catalysed by glucokinase, and then metabolism of the sugar. In β cells, oxidative glycolysis dominates with formation of pyruvate. An important feature of these cells, enabling increased generation of ATP, is anaplerotic metabolism of pyruvate. Conversion of pyruvate to lactate is small due to very low activity of lactate dehydrogenase [61]. This is accompanied by low expression of monocarboxylate transporter in the plasma membrane [27]. As a consequence, pyruvate generated by glycolysis is channelled to the mitochondria, being a substrate to the tricarboxylic acid cycle. Additionally, mitochondrial activity of pyruvate carboxylase, which catalyses conversion of pyruvate to oxaloacetate, is relatively high. This enables formation of citric acid cycle intermediates. The increased capacity of the citric acid cycle leads to the formation of reducing equivalents and to the increased generation of ATP [25, 40, 54, 77]. This is followed by a rise in ATP/ADP ratio, the closure of ATP-dependent K^+^ channels, depolarisation of the plasma membrane, opening of voltage-dependent Ca^2+^ channels and a rise in intracellular Ca^2+^ concentration. The latter effect triggers insulin exocytosis. Each step of this stimulus-secretion coupling is necessary to induce insulin release. Moreover, other intracellular signals are generated to maintain and potentiate the insulin-secretory response of β cells to glucose [25, 30, 54, 69]. Glucose-induced insulin secretion involves the triggering and the amplifying pathway. Both these pathways are energy-dependent [25, 47]. In line with this, an inhibition of processes associated with energy formation is well established to suppress hormone release [28, 40, 66]. All these data confirm the crucial role of ATP formation for glucose-induced insulin secretion.

**Fig. 1:**
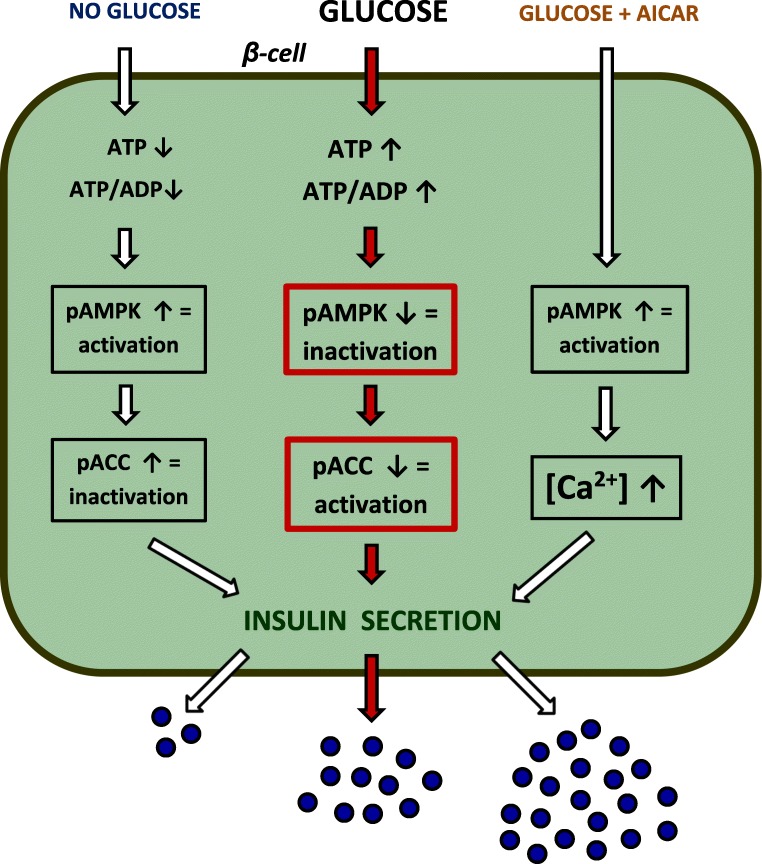
Effects of glucose alone and glucose in the presence of AICAR (an activator of AMPK) on AMPK and ACC activity in pancreatic β cells and the resulting changes in insulin secretion

Results of in vitro studies clearly show that elevated glucose levels are associated with a clear-cut reduction of the Thr172 phosphorylation level on the activation loop of the α-subunit of AMPK in β cells and the resulting inhibition of AMPK (Table 1, Fig. 1). This effect was found in pancreatic islets of rat, mouse and also human [3, 13, 40, 73]. In spite of some species differences related to β cell biology [41, 63, 69], the results regarding AMPK indicate that the influence of glucose is similar in rodent and human islets.

**Table 1:** Effects of glucose on AMPK, ACC and insulin secretion in pancreatic islets

Experimental conditions	Changes in AMPK and ACC	Insulin secretion	Reference
Rat islets, 3 and 17 mM glucose compared with 0 mM glucose, 60 min	AMPK activity reduced	Increased	32
Rat islets, 8.3 and 16.7 mM glucose compared with 3.3 mM glucose, 60 min	pAMPK reduced AMPK unchanged pACC reduced ACC unchanged	Increased	3
Rat islets, 3 and 17 mM glucose compared with 0 mM glucose, 16 h	AMPK activity reduced	Non-studied	32
Rat islets, 8.3, 11.1 and 16.7 mM glucose compared with 3.3 mM glucose, 20 h	pAMPK reduced AMPK unchanged pACC reduced ACC unchanged	Increased	13
Mouse islets, 8 or 14 mM glucose compared with 5 mM glucose, 30 min	AMPK activity reduced	Increased	73
Human islets, 6.7 mM glucose compared with 1 mM glucose, 60 min	pAMPK reduced	Increased	48
Human islets, 3 and 17 mM glucose compared with 0 mM glucose, 16 h	AMPK activity reduced	Non-studied	32

*AMPK* AMP-activated protein kinase, *ACC* acetyl-CoA carboxylase

Insulin secretion is a dynamic process and changes rapidly in response to various regulatory agents. In this context, the phosphorylation level of AMPKα (Thr172) was shown to be reduced already after short-term (30–60 min) exposure of pancreatic islets to glucose [3, 32, 73]. However, in the case of more prolonged (16–20 h) incubations of islets with increased concentrations of glucose, pAMPK is also reduced [13, 32]. This points to a similar effect of elevated glucose levels on pAMPK within minutes and hours.

Changes in the AMPK system induced by glucose in the insulin-secreting cells appear to include mostly the phosphorylation level and activity of AMPK, but not expression. This is evidenced by results showing that elevated glucose levels markedly reduce pAMPK, even after short-term treatment. However, intracellular expression of AMPK is not significantly influenced by glucose, neither in the case of short-term nor prolonged (16–20 h) exposure (Table 1).

These data clearly show that the rise in glucose levels, associated with increased insulin secretion, is accompanied by a concomitant inhibition of AMPK. It should be, however, mentioned that very low concentrations of glucose, insufficient to induce insulin release, are also capable of reducing pAMPK, compared with effects observed in the absence of this sugar (Table 1) [32].

Apart from glucose, other compounds influencing insulin secretion were shown to affect islet AMPK. Such an action was demonstrated in mouse and human islets subjected to glutamine and BCH (2-aminobicyclo-[1, 2] heptane-2-carboxylic acid). Pancreatic islets exposed to these compounds displayed markedly reduced phosphorylation of AMPK, compared with effects observed in control islets [51]. In pancreatic β cells, BCH is an allosteric activator of glutamate dehydrogenase, and the combination of glutamine with BCH has a clear-cut insulinotropic effect. The influence of glutamine and BCH on β cell pAMPK is an important finding, given differences in the metabolism of these compounds and glucose. Moreover, this indicates that glucose is not the sole metabolisable secretagogue, which is capable of affecting AMPK in β cells. However, glutamine induces insulin secretion only in the presence of pharmacological activation of glutamate dehydrogenase [42, 62] or under conditions of hyperactivity of this enzyme [21]. Therefore, this effect is without physiological relevance. On the other hand, it was shown that inhibition of glutamate dehydrogenase (by epigallocatechin-3-gallate) in mouse and human pancreatic islets is associated with activation of AMPK and a concomitant decrease in insulin secretion [51].

The regulatory influence on islet AMPK was also shown for adiponectin. This is an adipocyte-derived hormone, which positively affects several processes, also functioning of pancreatic islets [16]. Adiponectin receptors (AdipoR1 and AdipoR2) are expressed in human and rodent islet cells. Adiponectin was shown to enhance glucose-induced insulin secretion from rat islets with a concomitant increase in the phosphorylation level of AMPK. Both effects are particularly seen in the presence of higher glucose concentrations and upon a few hour exposure to adiponectin [22].

The increase in glucose concentrations and the resulting potentiation of insulin secretion is strongly associated with metabolic events in β cells and with changes in activities of several enzymes. One of the pivotal intracellular targets of AMPK is acetyl-CoA carboxylase (ACC). ACC is expressed in different kinds of cells and catalyses conversion of acetyl-CoA to malonyl-CoA. It is also present in the appreciable levels in the insulin-secreting cells [6]. Under conditions of low energy status, AMPK undergoes activation and phosphorylates ACC. This phosphorylation represses ACC. However, in response to increased glucose levels, AMPKα is dephosphorylated (and thereby inhibited) and does not phosphorylate ACC (Table 1, Fig. 1). This leads to the activation of ACC. Moreover, catabolism of glucose (and some other nutrients) markedly elevates concentrations of citrate, which serves in β cells not only for the formation of malonyl-CoA, but also allosterically activates ACC [6, 37]. This metabolic upregulation is in concert with the regulatory impact of AMPK on ACC. Finally, the activation of ACC results in increased generation of malonyl-CoA in β cells, which reduces β-oxidation (via inhibition of CPT-1) and simultaneously promotes formation of lipid signalling molecules [54].

Apart from ACC, SIRT1 is another important target of AMPK in various tissues, including liver, skeletal muscles and adipose tissue [18]. SIRT1 is an NAD^+^-dependent protein deacetylase, which is activated in response to changes in intracellular energy status. A decrease in the NAD/NADH ratio suppresses activity of SIRT1 [39]. Both AMPK and SIRT1 interact each other [18, 52, 79]. SIRT1 is expressed in pancreatic islets [5, 46], and its regulatory function in β cells is associated with changes in expression of several genes [33]. SIRT1 represses, among others, expression of uncoupling protein-2 (UCP2) [5]. It is also known that SIRT1-deficient islets display abated insulin-secretory response to glucose [39, 50]. Moreover, human pancreatic islets exposed to resveratrol (3,5,4′-trihydroxystilbene) were demonstrated to release more insulin compared with control islets. This increase was accompanied by upregulation of some genes (GLUT2 and glucokinase) and was dependent on the presence of active SIRT1. These changes were, however, seen after prolonged (24 h) incubation [72]. Moreover, intracellular effects of resveratrol are pleiotropic and involve not only activation of SIRT1. The direct link between AMPK and SIRT1, especially in the context of regulation of insulin release, had never been shown. This allows to suppose that the AMPK/SIRT1 pathway is not involved in the short-term regulation of insulin secretion.

The relevance of the AMPK system in β cells is often studied using AICAR (AICA riboside, 5-aminoimidazole-4-carboxamide ribonucleotide), which is a pharmacological activator of AMPK. This compound undergoes an intracellular phosphorylation by adenosine kinase to ZMP (5-aminoimidazole-4-carboxamide ribonucleotide 5′-monophosphate). Then, ZMP, being an analogue of AMP, binds to the CBS domains on γ-subunit of AMPK. This is followed by an allosteric activation and phosphorylation of Thr172. The concentrations of AICAR needed to effectively activate AMPK in islet cells are usually between 0.1 and 1 mM [2, 14, 60, 73].

Akkan and Malaisse have reported that pharmacological induction of AMPK by AICAR significantly influences the insulin-secretory response of rat islets to glucose [2]. Insulin release from the isolated islets was shown to be markedly elevated in the presence of glucose and AICAR, compared with secretion elicited by glucose alone. This potentiatory effect has been confirmed by several other studies (Table 2, Fig. 1). The increase in insulin secretion due to the activation of AMPK by AICAR occurs over a broad range of glucose concentrations [2, 14, 73]. However, AICAR fails to affect hormone release at low glucose, and also in the absence of this sugar [2, 14]. This indicates that the presence of glucose (or other secretagogue) in a concentration that stimulates secretion is a prerequisite for the occurrence of the potentiatory effect resulting from activation of AMPK. Apart from glucose, such an effect was observed in the case of α-ketoisocaproate (α-KIC), an intermediate compound in the metabolism of leucine. The insulin-secretory response of pancreatic islets to α-ketoisocaproate was shown to be significantly elevated in the presence of AICAR [2, 43].

**Table 2:** Effects of pharmacological activation of AMPK by AICAR on insulin secretion from pancreatic islets

Experimental conditions	AICAR	Insulin secretion	Reference
Rat islets, 5.6 to 20 mM glucose	0.1–1 mM	Increased	[2]
Rat islets, 0 and 2.8 mM glucose	0.1–1 mM	Unchanged	[2]
Rat islets, 3.3 and 5.5 mM glucose	1 mM, 30 or 60 min	Increased	[60]
Mouse islets, 8, 10 and 15 mM glucose	0.5 mM, 60 min	Increased	[14]
Mouse islets, 3 and 6 mM glucose	0.5 mM, 60 min	Increased	[14]
Mouse islets, 5, 8 and 14 mM glucose	0.3 mM, preincubation with 2 mM glucose, 60 min, incubation 30 min	Increased	[73]

*AMPK* AMP-activated protein kinase, *AICAR* AMPK activator

Lack of effects of AMPK pharmacological activation on insulin release in the absence of glucose is in line with physiological conditions. At low concentrations of this secretagogue, insufficient to induce insulin secretion, intracellular transport and metabolism of this sugar in β cells is very limited, thus preventing exaggerated supply of the hormone.

Results of studies with the use of perfused rat pancreas have confirmed that glucose-induced insulin output is significantly enhanced as a result of AMPK activation by AICAR [2, 7]. The influence of AICAR on AMPK, and the resulting potentiation of insulin secretion, reveals already within 2–3 min after the inclusion of this compound to the perfusion medium [7]. Insulin release stimulated by glucose is usually biphasic, which is clearly seen at higher concentrations of the secretagogue. The pharmacological AMPK induction was shown to markedly increase both the first and the second phase of secretion [7].

The maximal increase of glucose-induced insulin secretion from rat and mouse islets, resulting from the activation of AMPK by AICAR, is about 50% [14, 60], compared with secretion elicited by glucose alone. It should be, however, emphasised that these results were shown in vitro, in the absence of other agents that modulate the secretory function of β cells. Therefore, effects in vivo are difficult to assess.

## Mechanism of AMPK action

Elevated glucose levels increase insulin secretion with a simultaneous inhibition of AMPK in β cells. However, AICAR is capable of preventing glucose-induced AMPK inhibition and induces a further rise in insulin secretion [32, 73]. Glucose-induced AMPK inhibition may be restored to the level observed in the islet cells deprived of glucose [32].

The data on the mechanisms of AMPK action in β cells are not fully unequivocal. The potentiatory effects on glucose-induced insulin secretion, resulting from pharmacological activation of AMPK, may involve different steps of the stimulus-secretion coupling. Under physiological conditions, glucose metabolism in β cells is a prerequisite to increase their secretory capacity. However, some studies have shown that the effect of AICAR on glucose-induced insulin secretion from pancreatic islets is accompanied neither by changes in glucose oxidation nor utilisation [43]. Moreover, potentiatory effect of AICAR on insulin release was found to be without any influence on mitochondrial membrane potential and NAD(P)H autofluorescence, indicating that oxidative phosphorylation is unchanged by this compound [60].

Other studies indicate that AMPK influences the electrical activity of the insulin-secreting cells [36, 60]. The rise in the electrical activity of the β cell membrane, induced by glucose alone, was reported to be additionally enhanced in the presence of AICAR. This effect was due to the inhibition of the K^+^ ATP current in β cells. It is suggested that this inhibition does not result from the direct interaction of AICAR with K_ATP_ channel [60, 73]. On the other hand, in pancreatic islets of mice lacking the K_ATP_ channel subunit sulfonylurea receptor 1 (Sur1ˉ^/^ˉ), AICAR activates AMPK and potentiates glucose-induced insulin secretion. This suggests that the effects are partially K_ATP_ channel-independent [73].

More consistent results were obtained with respect to the changes in AMPK activity and cytosolic Ca^2+^ concentrations ([Ca^2+^]_c_). Malaisse et al. have shown that exposure of rat islets to AICAR increases Ca^2+^ uptake by islet cells [43]. The influence AMPK of activation on increased Ca^2+^ uptake was confirmed by many other studies [48, 60, 73]. Given the role of Ca^2+^ in the process of insulin secretion, this finding partially explains the increased hormone secretion resulting from activation of AMPK. A rise in [Ca^2+^]_c_ induces insulin exocytosis, and also largely enhances intracellular metabolism (some mitochondrial enzymes are subjected to control by Ca^2+^; therefore, mitochondrial oxidative metabolism is stimulated in response to Ca^2+^) [59]. In parallel with results concerning insulin secretion, pharmacological AMPK activation elevates [Ca^2+^]_c_ in the presence of stimulatory concentrations of glucose. This rise is, however, not observed in the absence of glucose [48, 73]. These results clearly point to the role of increased [Ca^2+^]_c_ for potentiation of insulin release under conditions of AMPK pharmacological activation (Fig. 1).

Apart from modulating insulin secretion, AMPK is known to affect some other processes related to β cell biology. Results of the recent studies show that AMPK is involved in the regulation of the expression of several genes in β cells, including insulin gene [44, 57]. This effect is mediated by micro RNAs (miRNAs). Micro RNAs are small, non-coding molecules that silence gene expression. Glucose-mediated changes in phosphorylation level of AMPK regulate miRNAs expression and thereby affect expression of genes in pancreatic β cells. In the insulin-secreting cells, miR-184 is one of the pivotal regulators. Its expression is downregulated by elevated glucose concentrations. The influence of glucose on miR-184 expression has been recently shown to be mediated by AMPK. It was demonstrated that depletion of AMPK largely impairs expression of miR-184 in β cells. However, an increase in AMPK activity is associated with upregulation of miR-184. This effect was revealed in a murine cell line (MIN6), in mouse pancreatic islets, and also in human islets [44]. Apart from regulatory effects of glucose, pharmacological activation or inhibition of AMPK was also shown to affect gene expression. These new findings indicate the possibility of regulation of gene expression in pancreatic β cells via pharmacological AMPK modulation in these cells.

## AMPK under pathological conditions

The insulin-secreting cells are still subjected to changes in the supply of energy substrates, primarily glucose and fatty acids. It is well established that chronically elevated levels of glucose (glucotoxicity) and/or lipids (lipotoxicity) negatively affect energy homeostasis of β cells. This leads to metabolic disturbances and to a gradual failure of the insulin-secreting cells [53, 54]. This phenomenon is observed in type 2 diabetes. Therefore, an appropriate regulation of metabolic pathways is essential to preserve cellular functionality. In this context, β cell AMPK could be a target for pharmacological modulation, especially under pathological conditions.

Del Guerra et al. have described several functional and molecular defects, which develop in pancreatic islets of humans with type 2 diabetes [12]. These islets release less insulin in response to glucose, also display diminished glucose oxidation, reduced insulin gene expression and increased oxidative stress. These pathological changes were shown to be accompanied by downregulation of AMPK in islet cells.

In pancreatic islets of healthy subjects, elevated glucose concentrations are associated with the suppression of pAMPK. This regulatory effect is, however, attenuated under some pathological conditions. Such an attenuation was shown in mice fed a high-fat diet. In normal mice, pancreatic islets subjected to high glucose displayed markedly reduced pAMPK (Thr172). However, in animals on a high-fat diet, the influence of elevated glucose concentrations on the phosphorylation level of AMPK was much less marked [49]. According to these results, the link between glucose and pAMPK was demonstrated to be also disturbed in pancreatic islets of *ob/ob* mice. This model is characterised by obesity due to mutations in leptin gene. In islets derived from both normal and *ob/ob* mice, high glucose decreased AMPK phosphorylation/activity. However, the impact of glucose was much weaker in the latter animals [49].

It is also known that loss of AMPKα2 in pancreatic β cells is associated with diminished insulin-secretory response to glucose and with reduced level of UCP2. This indicates that the proper AMPK expression/activity in these cells is essential to maintain normal glucose sensing [4]. Given that the antioxidtive defence status in β cells is low [35], relatively high UCP2 levels in these cells are vital to reduce the mitochondrial membrane potential and to attenuate production of reactive oxygen species [1, 28].

On the other hand, over-expression of constitutively active AMPK (AMPK CA) in human and rodent islets is associated with several defects. Pancreatic islets with over-expression of AMPK CA secrete less insulin [56] and are also characterised by reduced glucose oxidation. Over-expression of AMPK in islet cells may be without significant influence on insulin content, however, may increase apoptosis and negatively affect β cell functionality [32, 56]. It was demonstrated that transplantation of pancreatic islets with over-expression of AMPK CA into mice treated with streptozotocin (which destroys selectively pancreatic β cells) [34, 67] improves glycemic control less effectively than islets derived from normal mice [56]. These data indicate that both downregulation or over-expression of AMPK in pancreatic β cells are associated with functional defects of β cells.

Islet AMPK may play a regulatory role in β cells in the case of exaggerated supply of free fatty acids. Free fatty acids participate in the regulation of insulin secretion, since their short-term action at low concentrations of glucose is important to maintain β cell functionality [54]. However, prolonged exposure leads to lipotoxicity and to β cell failure [38]. Wang et al. showed that treatment of rat islets with palmitate increases both phosphorylation level of AMPK and glucose-induced insulin secretion [74]. It is suggested that AMPK activation as a result of exposure to fatty acids and the resulting metabolic changes in β cells may have protective role to prevent exaggerated lipid accumulation and cellular dysfunction [54, 74].

Pharmacological activation of AMPK can protect pancreatic islet cells against glucolipotoxicity. This effect was shown in islets of normal rats maintained for 1–3 days under glucolipotoxic conditions. The functional defects developing in pancreatic islets were markedly attenuated as a result of pharmacological induction of AMPK. In the presence of pharmacological activator, the phosphorylation level of AMPK was restored, and also the expression of several genes was normalised [29].

## Conclusions

Results of studies using cell lines addressing regulatory function of AMPK in the insulin secretion process are not conclusive. Some studies demonstrate that AMPK pharmacological activation inhibits glucose-induced insulin release [9, 17, 60, 71], but the other indicate stimulatory effects [15, 31, 80] or lack of changes in insulin release [15]. However, experimental findings arising from studies using isolated pancreatic islets, β cells and isolated pancreas are much more coherent. They show that both over-expression or under-expression of AMPK significantly disturb insulin secretion, which indicates that appropriate levels of AMPK are necessary to preserve secretory function of β cells. It is also well established that the rise in glucose concentrations potentiates insulin release and is associated with reduced AMPK phosphorylation/activity. This inhibition of AMPK leads to the decreased phosphorylation and activation of ACC. It is also known that pharmacological induction of AMPK in the presence of glucose markedly enhances the insulin-secretory capacity of β cells. This effect is largely due to a rise in the intracellular Ca^2+^ levels. The potentiation of insulin secretion is seen already within minutes after activation of AMPK. This is a relevant finding, given possibility of short-term regulation of insulin secretion via pharmacological AMPK modulation.

AMPK is also involved in the regulation of expression of several genes in β cells, including insulin gene. Moreover, pharmacological modulation of AMPK was shown to positively affect β cells under some pathological conditions.

However, more studies are required to fully clarify the mechanism of AMPK action in pancreatic β cells under physiological conditions. Little is known about the link between activation or inhibition of AMPK and the resulting metabolic changes in β cells. Elucidation of this question could be helpful in the potential use of selective AMPK modulators. Moreover, action of AMPK in β cells under pathological conditions is still poorly explained. This is a relevant issue, given that pharmacological regulation of AMPK could effectively improve β cell function.
